# Therapeutic effects and safety of resveratrol for lung cancer: an updated preclinical systematic review and meta-analysis

**DOI:** 10.3389/fnut.2025.1644538

**Published:** 2025-08-29

**Authors:** Xiang Xiao, Xuanyu Wu, Wenyuan Li, Fengming You, Jing Guo

**Affiliations:** ^1^Hospital of Chengdu University of Traditional Chinese Medicine, Chengdu University of Traditional Chinese Medicine, Chengdu, China; ^2^Evidence-Based Traditional Chinese Medicine Center of Sichuan Province, Chengdu, China; ^3^Cancer Institute, Chengdu University of Traditional Chinese Medicine, Chengdu, China

**Keywords:** lung cancer, resveratrol, preclinical evidence, systematic review, meta-analysis

## Abstract

**Background:**

Lung cancer (LC) is the most common cause of cancer-related death worldwide, while there are limited treatment methods. Resveratrol (RESV), a natural food-derived compound, has attracted attention around the world for its anti-LC effects. However, little is known about the efficacy and safety of RESV for LC.

**Purpose:**

This study aimed to provide preclinical evidence for the efficacy and safety of RESV for LC, and to find the optimal dose and duration.

**Methods:**

*In vivo* studies of RESV against LC, published before 24 July 2024, were retrieved from PubMed, Embase, Web of Science, and Cochrane Library. The CAMARADES checklist was used to assess study quality. Primary outcomes were tumor volume and tumor weight. Secondary outcomes included body weight, lung metastases number, and the apoptotic cell proportion. Statistical analysis was performed using RevMan 5.3 and Stata 16.0. Dose–duration–effect model was conducted to determine the optimal dose and duration, and the toxicology of RESV was predicted through the ProTox 3.0 platform.

**Results:**

A total of 23 studies involving 425 animals were included. The methodological quality of included studies was medium-to-low. RESV significantly reduced tumor volume, tumor weight, and lung metastases number, and increased apoptotic cell proportion, while having no effect on body weight. High heterogeneity was observed, and subgroup analysis suggested that the heterogeneity was partly attributed to the dose of RESV. The optimal dose and duration of RESV were 30–100 mg/kg and 25–28 days, respectively. The median lethal dose of RESV was 1,560 mg/kg.

**Conclusion:**

RESV demonstrated a significant inhibitory effect on LC *in vivo*. However, the lower research quality and high heterogeneity call for more high-quality preclinical studies to be conducted. Before achieving clinical translational research on RESV, the problem of low bioavailability of RESV needs to be solved.

## Introduction

Lung cancer (LC) led to 1,817,172 deaths in 2022, and remains the most common cause of cancer-related death (18.7% of total deaths), resulting in an enormous global social and economic burden (1). The disastrous prognosis of LC is largely due to factors, including late-stage diagnosis (2), acquired drug resistance, and low treatment tolerance (3), which significantly impede effective treatment and contribute to the high mortality rate. Indeed, various treatment strategies, including surgery, chemotherapy, radiotherapy, targeted therapy, and immunotherapy, have moderately improved survival outcomes in patients with LC. Yet, the overall prognosis remains poor, and the estimated 5-year survival was only 26.4% (4). Additionally, existing pharmacological treatments exhibit significant limitations. Chemotherapy is associated with notable adverse effects, including neurotoxicity, gastrointestinal disturbances, and cardiovascular toxicity (5). Targeted therapies are applicable to a specific subset of patients and are prone to inducing adverse reactions such as irreversible pulmonary fibrosis (6). Immunotherapy is effective for only a limited population and is frequently accompanied by the development of acquired resistance (7). Consequently, there is an urgent need to identify and develop efficacious, tolerable, and safe therapeutic strategies for LC.

**Figure 1:**
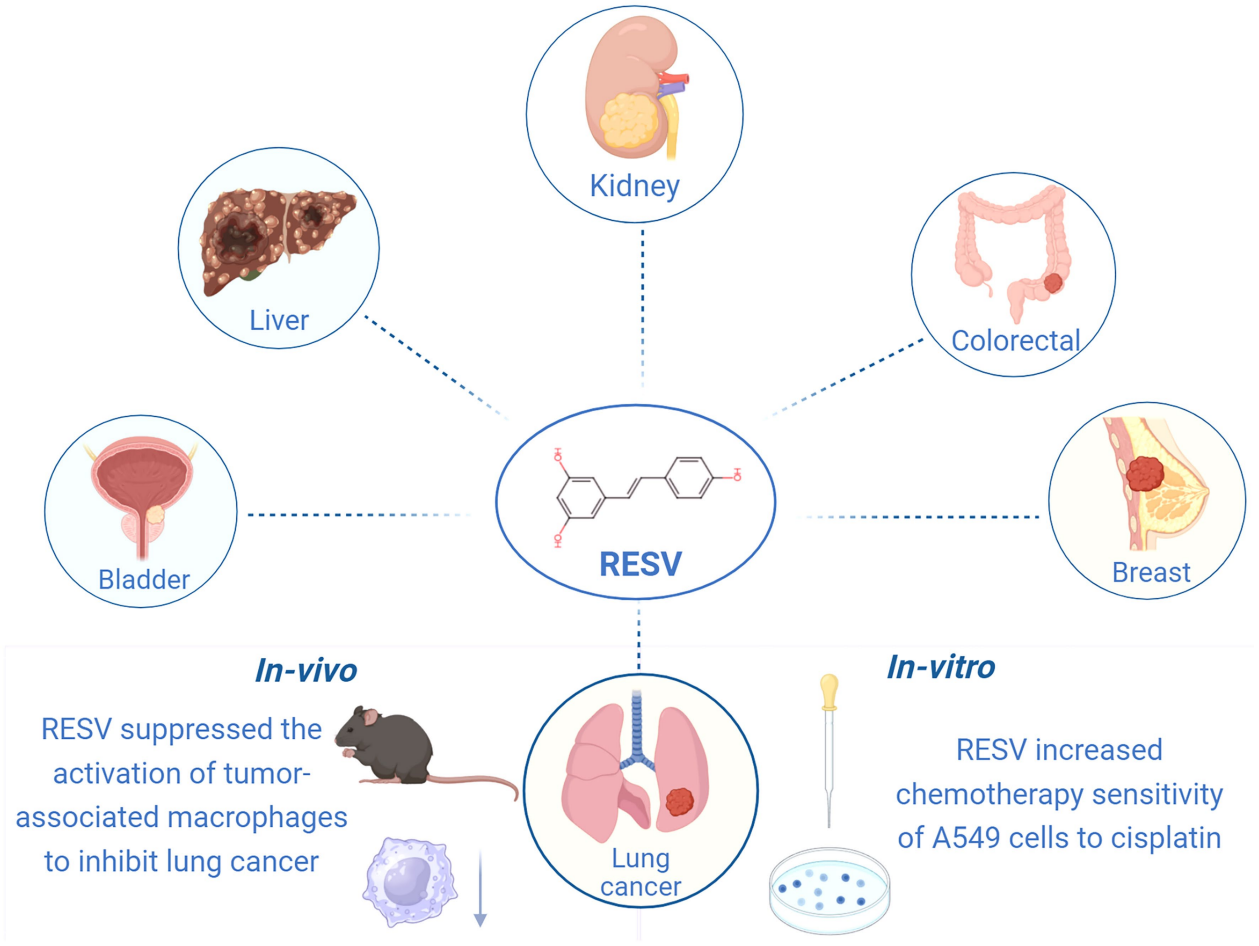
Anti-cancer effects of RESV (created using BioRender.com).

Identifying compounds with anticancer activity in natural foods has been a topic of interest for years. Resveratrol (RESV), a phenolic compound found in many plants such as grapes, blueberries, and peanuts, exhibits various pharmacological and biological activities, including anti-aging, anti-inflammatory, antioxidant, antifibrotic, and anticancer effects (8, 9). Indeed, RESV has shown potential in treating a wide range of cancers, including lung, breast, colorectal, renal, liver, and bladder cancers (10–15) (Figure 1). In clinical trials, RESV has demonstrated promising properties for colorectal, breast, and prostate cancers (16). Unfortunately, no clinical study has yet confirmed whether RESV is effective in patients with LC, although numerous *in vivo* and *in vitro* studies have explored its anti-LC effects. *In vitro*, RESV treatment increased the chemical sensitivity of A549 cells to cisplatin (10). *In vivo,* RESV can inhibit LC progression by suppressing the activation of tumor-associated macrophages (17). Nevertheless, the protective effect of RESV against LC in animals has not been systematically reviewed, and its mechanism remains unknown.

A systematic review and meta-analysis of preclinical studies provides valuable insights into the reliability of preclinical studies and facilitates the transition from animal to clinical trials (18). The absence of clinical trials investigating RESV for LC treatment currently precludes robust evaluation of its therapeutic efficacy and safety in human patients. Nevertheless, a comprehensive meta-analysis of preclinical studies may yield valuable therapeutic insights and critical preliminary guidance for future clinical translation. A meta-analysis published in 2016 reported RESV significantly reduced the incidence of LC by 36% *in vivo* (19). With the publication of numerous studies focusing on RESV against LC, it is necessary to conduct an updated meta-analysis to improve the accuracy of estimated effects. Herein, we systematically reviewed the effects of RESV in LC animals in order to provide preclinical evidence for subsequent clinical trials. We hope RESV may become a promising natural treatment for LC in the future.

## Materials and methods

### Study registration

This study followed the Preferred Reporting Items for Systematic Reviews and Meta-Analyses (PRISMA) guideline (20), and has been registered on PROSPERO (CRD42024569393).

### Search strategy

PubMed, Embase, Web of Science, and the Cochrane Library were searched by two authors (Xiang Xiao and Xuanyu Wu) to identify relevant studies. The search period was from the establishment of the database to 24 July 2024. The search was carried out by using MeSH combined with free words (Supplementary Table 1). Additionally, we searched the references of included studies to collect other potential studies.

### Study selection

Two authors (Xiang Xiao and Wenyuan Li) selected studies following the PRISMA guidelines using EndNote X9 software. Selection results were cross-checked to ensure consistency, and a third investigator (Jing Guo) was consulted to resolve disagreements. We screened titles and abstracts to exclude irrelevant and non-English studies after duplicate papers were eliminated through electronic and manual-based steps. Full texts of the remaining studies were then reviewed to confirm final eligibility.

Based on the PICOS principle, the inclusion criteria were as follows: (1) participants (animals): LC model animals, including orthotopic tumors and ectopic transplanted tumors, without limitation of species, sex, or age; (2) intervention: the intervention group was treated with RESV, and the dose and duration were clarified; (3) comparator: the control group was treated with placebo or saline; (4) outcome measure: the primary outcomes were tumor volume and tumor weight, and the secondary outcomes were lung metastases number, body weight, and apoptotic cell proportion; and (5) study design: only *in vivo* animal studies with separate treatment groups were eligible.

The exclusion criteria were as follows: (1) studies that were not *in vivo* animal studies, such as clinical studies, *in vitro* studies, *in silico* studies, reviews, letters, conference papers, abstracts, and editorials; (2) studies with missing data; (3) studies in which the intervention group received RESV derivatives or analogs; and (4) studies without any pre-set outcomes.

### Data extraction

Two authors (Xiang Xiao and Xuanyu Wu) extracted the following information independently using Excel 2021 software: (1) article information, including first author’s name, publication year, and country; (2) animal information, including species, sex, age, and weight; (3) modeling information, including modeling method, drug/cell, route, dose, duration, and anesthetic; (4) intervention information, including dose, duration, and route; (5) sample size of both intervention and control groups; and (6) mean and standard deviation (SD) of outcome measures (final time point result). The WebPlotDigitizer^1^ was employed to measure graphic values. The *p*-values not reported in studies were calculated using the independent samples t-test (summarized data) function in the SPSS 26.0 software. An investigator (Xiang Xiao) contacted the corresponding authors to get missing data.

### Risk of bias assessment

The 10-item CAMARADES checklist (21) was employed by two authors (Xiang Xiao and Wenyuan Li) independently to evaluate the quality of included studies. The assessment criteria were as follows: (1) peer reviewed publication; (2) control of temperature; (3) random allocation to treatment or control; (4) blinded induction of ischemia; (5) blinded assessment of outcome; (6) use of anesthetic without significant intrinsic neuroprotective activity; (7) animal model (aged, diabetic, or hypertensive); (8) sample size calculation; (9) compliance with animal welfare regulations; and (10) statement of potential conflict of interests. Each item was evaluated at “high,” “low,” or “unclear” risk based on the information reported in the full text and Supplementary materials.

### Dose–duration–effect 3D model

The Origin 2021 software was used to display 3D models of the dose and duration of RESV and the primary outcome measures (tumor volume and tumor weight).

### RESV toxicology prediction

The toxicity assessment of RESV was insufficient in the included studies. Herein, ProTox 3.0,^2^ an online platform, was employed to predict the toxicology of RESV. The organ toxicity, carcinogenicity, immunotoxicity, mutagenicity, cytotoxicity, blood–brain barrier (BBB) permeability, ecotoxicity, clinical toxicity, and nutritional toxicity were predicted.

### Statistics analysis

The RevMan 5.3 and Stata 16.0 were used for meta-analysis. The weighted mean difference (WMD) and 95% confidence interval (CI) were used when comparing continuous variables with consistent measurement methods and units across studies; otherwise, the standardized mean difference (SMD) and 95% CI were applied.

Heterogeneity was assessed using the *I^2^* statistic and Cochrane’s *Q* test. Heterogeneity was graded as high (*I*^2^ > 75%), moderate (50% ≤ *I*^2^ ≤ 75%), or low (*I*^2^ < 50%). For Cochrane’s *Q* test, a *p-*value < 0.05 indicates significant heterogeneity. When high or moderate grade heterogeneity was observed, we first performed sensitivity analysis by sequentially removing each trial to determine if any single study contributed to the heterogeneity. If substantial heterogeneity in tumor volume persisted and its source remained unexplained after sensitivity analysis, subgroup analysis or meta-regression was performed to explore potential causes. Subgroup analysis was stratified by the dose of RESV (22). Meta-regression analysis identified eight variables as potential sources of heterogeneity: publication year, dose, duration, administration, modeling method, species, gender, and region. Variables with a *p-*value < 0.05 were considered significant contributors to heterogeneity. For low-grade heterogeneity, we used a fixed-effect model to combine effect sizes; otherwise, a random-effects model was employed. Funnel plots and Egger’s regression test were conducted to explore publication bias when there were more than 10 studies.

## Results

### Search and selection results

**Figure 2:**
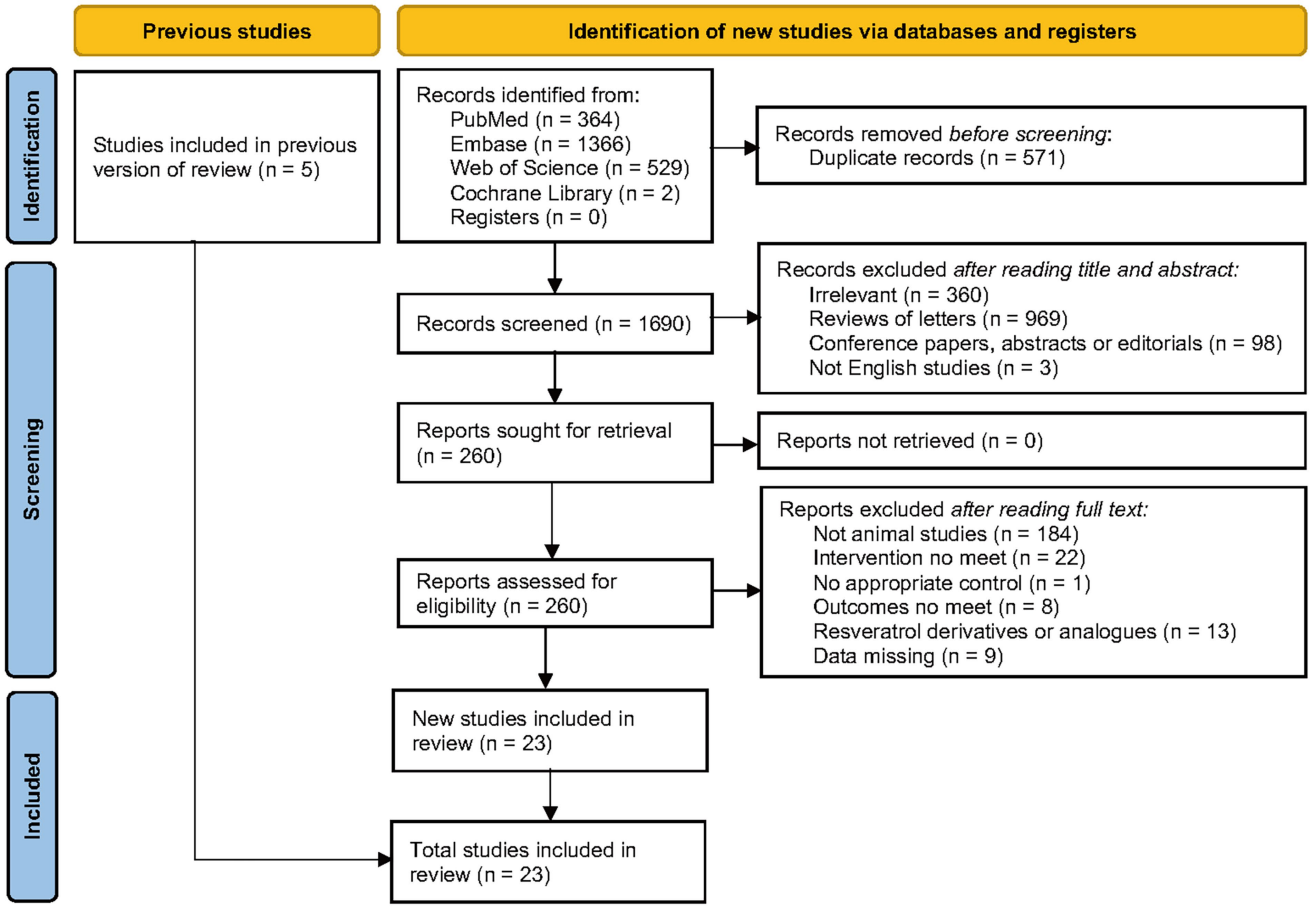
Flow chart of the study selection process.

A total of 2,261 records were obtained through database screening, and 1,690 records were obtained after eliminating duplicates. After reading titles and abstracts, 260 records remained. After reviewing the full text of the 260 articles, 23 studies were ultimately included for meta-analysis. For different subgroups present in a study, we treated them as different experiments, and among the 23 studies, there were 30 experiments (Figure 2).

### Characteristics of included studies

Table 1 shows basic characteristics of the 23 included studies. These studies were published from 2001 to 2024. There were 211 and 214 animals in the intervention and control groups, respectively. In total, 7 studies used BALB/c mice (23–29), 6 studies used C57BL/6 J mice (17, 30–34), 5 studies used nude mice (35–39), 2 studies used laka mice (40, 41), 1 study used severe combined immunodeficient mice (42), 1 study used A/J mice (43), and 1 study used the Rowett nude rat (44). A total of 10 studies (23–25, 28, 30, 31, 35, 37, 42, 43) used female mice, and 7 studies (29, 33–35, 40, 41, 44) used male mice, while the other 7 studies (17, 26, 27, 32, 36, 38, 39) did not report the sex of animals. In addition, 4 studies established mouse models of orthotopic xenograft (40, 41, 43, 44), while the remaining studies used ectopic transplanted tumor mouse models. The dose range of RESV was from 0.23 to 3,000 mg/kg. The duration ranged from 6 to 84 days. The routes of RESV included oral gavage, intratumor injection, intranasal infusion, intravenous injection, and intraperitoneal injection.

**Table 1:** Basic characteristics of the included studies.

Study	Region	Species (sex, age, *n* = experimental/control group)	Weight (g) (experimental/control group)	Model method (drug/cell, route, dose, duration)	Anesthetic	Intervention (experimental/control group, route)	Dose, duration	Outcomes	*p*-value
Kimura et al. (30)	Japan	C57BL/6 mice (Female, 5, 7/7)	18.8 ± 0.40/18.1 ± 2.51	HTT (LLC, Sc, 5 × 10^5^, once)	NA	RESV+LLC/LLC, Ip	0.6 mg/kg, QD, 21 days	1; 2; 3; 4	1. P > 0.05; 2. *p* < 0.05; 3. *p* > 0.05; 4. *p* < 0.05
Kimura et al. (30)	Japan	C57BL/6 mice (Female, 5, 7/7)	18.5 ± 1.09/18.1 ± 2.51	HTT (LLC, Sc, 5 × 10^5^, once)	NA	RESV+LLC/LLC, Ip	2.5 mg/kg, QD, 21 days	1; 2; 3; 4	1. *p* > 0.05; 2. *p* < 0.05; 3. *p* > 0.05; 4. *p* < 0.05
Kimura et al. (30)	Japan	C57BL/6 mice (Female, 5, 7/7)	18.9 ± 0.74/18.1 ± 2.51	HTT (LLC, Sc, 5 × 10^5^, once)	NA	RESV+LLC/LLC, Ip	10 mg/kg, QD, 21 days	1; 2; 3; 4	1. *p* < 0.05; 2. *p* < 0.05 3. *p* > 0.05; 4. *p* < 0.05
Lee et al. (31)	Korea	C57BL/6 J mice (Female, 5, 6/6)	NA	HTT (LLC, Sc, 3 × 10^5^/100 μL, once)	NA	RESV+LLC/LLC, Ip	20 mg/kg, QD, 21 days	1; 2; 5	1. *p* < 0.001; 2. *p* < 0.01; 5. *p* < 0.05
Busquets et al. (32)	Spain	C57BL/6 mice (NA, 12, 6/6)	24.7 ± 2.45/25.7 ± 2.69	HTT (LLC, Im, 5 × 10^5^, once)	Ketamine and xylazine	RESV+LLC/LLC, Ip	5 mg/kg, QD, 15 days	1; 2; 3; 4	1. *p* > 0.05; 2. *p* > 0.05 3. *p* > 0.05; 4. *p* < 0.05
Busquets et al. (32)	Spain	C57BL/6 mice (NA, 12, 6/6)	23.9 ± 1.96/25.7 ± 2.69	HTT (LLC, Im, 5 × 10^5^, once)	Ketamine and xylazine	RESV+LLC/LLC, Ip	25 mg/kg, QD, 15 days	1; 2; 3; 4	1. *p* > 0.05; 2. *p* > 0.05 3. *p* > 0.05; 4. *p* < 0.05
Malhotra et al. (40)	India	Laka mice(Male, NA, 8/9)	18–20	*In situ* (BaP, Ip, 100 mg/kg, once)	Ether	RESV+BaP/BaP, Ga	5.7 mg/kg, Q2D, 35 days	3	3. *p* < 0.05
Zhao et al. (23)	China	BALB/c mice (Female, NA, 6/6)	18–22	HTT (SPC-A-1-CDDP, Sc, 1 × 10^8^, once)	NA	RESV+SPC-A-1-CDDP/SPC-A-1-CDDP, NA	1,000 mg/kg, QD, 28 days	1; 2	1. *p* < 0.05; 2. *p* < 0.05
Zhao et al. (23)	China	BALB/c mice (Female, NA, 6/6)	18–22	HTT (SPC-A-1-CDDP, Sc, 1 × 10^8^, once)	NA	RESV+SPC-A-1-CDDP/SPC-A-1-CDDP, NA	3,000 mg/kg, QD, 28 days	1; 2	1. *p* < 0.05; 2. *p* < 0.05
Malhotra et al. (41)	India	Laka mice(Male, NA, 8/9)	18–20	In situ (BaP, Ip, 100 mg/kg, once)	Ether	RESV+BaP/BaP, Ga	5.7 mg/kg, TW, 22 days	3	3. *p* < 0.001
Vetvicka et al. (24)	USA	BALB/c mice (Female, 8, 5/5)	NA	HTT (LLC, Im, 5 × 10^5^, once)	CO_2_	RESV+LLC/LLC, Ip	5 mg/kg, QD, 15 days	4	4. *p* > 0.05
Yin et al. (35)	China	Nude mice (Male & Female, 6–8, 6/6)	18–22	HTT (A549, Sc, 5 × 10^6^, once)	NA	RESV+A549/A549, Iv	15 mg/kg, QD, 15 days	1; 3	1. *p* < 0.05; 3. *p* > 0.05
Yin et al. (35)	China	Nude mice (Male and Female, 6–8, 6/6)	18–22	HTT (A549, Sc, 5 × 10^6^, once)	NA	RESV+A549/A549, Iv	30 mg/kg, QD, 15 days	1; 3	1. *p* < 0.01; 3. *p* > 0.05
Yin et al. (35)	China	Nude mice (Male and Female, 6–8, 6/6)	18–22	HTT (A549, Sc, 5 × 10^6^, once)	NA	RESV+A549/A549, Iv	60 mg/kg, QD, 15 days	1; 3	1. *p* < 0.01; 3. *p* > 0.05
Yu et al. (42)	China	SCI mice (Female, 4–6, 10/10)	NA	HTT (A549-FOXC2, Sc, 2 × 10^6^, once)	NA	RESV+A549-FOXC2/ A549-FOXC2, Ip	20 mg/kg, QD, 42 days	1	1. *p* < 0.05
Yu et al. (42)	China	SCI mice (Female, 4–6, 10/10)	NA	HTT (A549, Sc, 2 × 10^6^, once)	NA	RESV+A549/A549, Ip	20 mg/kg, QD, 42 days	1	1. *p* > 0.05
Bai et al. (36)	China	Nude mice (NA, 6–8, 15/15)	NA	HTT (H460, Sc, 3 × 10^7^/200 μl, once)	CO_2_	RESV+H460/H460, Ii	200 μl, B2D, 15 days	1; 2	1. *p* < 0.05; 2. *p* < 0.05
Vetvicka et al. (25)	USA	BALB/c mice (Female, 8, 5/5)	NA	HTT (LLC, Im, 5 × 10^6^, once)	CO_2_	RESV+LLC/LLC, Ga	5 mg/kg, QD, 14 days	4	4. *p* < 0.05
Li et al. (37)	China	Nude mice (Female, 5, 5/5)	NA	HTT (H460, Sc, 1 × 10^6^/100 μl, once)	NA	RESV+H460/H460, Ip	30 mg/kg, Q3D, 8 days	1; 2	1. *p* < 0.05; 2. *p* < 0.05
He et al. (38)	China	Nude mice (NA, NA, 5/5)	NA	HTT (A549, Iv, 1 × 10^7^/200 μl, once)	NA	RESV+A549/A549, Ip	10 mg/kg, QD, 14 days	4	4. *p* > 0.05
Sun et al. (17)	China	C57BL/6 mice (NA, 4–5, 5/5)	NA	HTT (LLC, Sc, 1 × 10^6^, once)	Ketamine and xylazine	RESV+LLC/LLC, Ip	100 mg/kg, QD, 28 days	1; 2	1. *p* < 0.01; 2. *p* < 0.01
Monteillier et al. (43)	Switzerland	A/J mice (Female, 5–6, 14/14)	14–16	In situ (NNK, Ip, 50 mg/kg, QW, 2doses)	NA	RESV+NNK/NNK, Nasal	80 mg/kg, TW, 26 days	1; 3	1. *p* < 0.01; 3. *p* < 0.05
Zhao et al. (33)	China	C57BL/6 mice (Male, 6–8, 6/6)	NA	HTT (LLC, Sc, 1 × 10^6^/200 μl, once)	NA	RESV+LLC/LLC, Ga	50 mg/kg, QD, 21 days	2	2. *p* < 0.01
Song et al. (26)	China	BALB/c mice (NA, 6–8, 8/8)	NA	HTT (HCC827, Sc, 1 × 10^6^/100 μl, once)	NA	RESV+HCC827/ HCC827, Iv	50 mg/kg, Q3D, 6 days	1; 2	1. *p* < 0.05; 2. *p* < 0.05
Zheng et al. (27)	China	BALB/c mice (NA, 4–6, 5/5)	NA	HTT (A549, Sc, 2 × 10^6^, once)	NA	RESV+A549/A549, Iv	15 mg/kg, Q3D, 15 days	1; 2	1. *p* < 0.001; 2. *p* > 0.05
Qin et al. (39)	China	Nude mice (NA, NA, 5/5)	NA	HTT (HCC827, Sc, 2 × 10^6^/200 μl, once)	NA	RESV+HCC827/ HCC827, Iv	0.23 mg/kg, 5 times/week, 25 days	1; 2	1. *p* < 0.01; 2. *p* > 0.05
Wang et al. (44)	China	Rowett nude rats (Male, 8, 8/8)	240–260	In situ (A549, Nasal, 2 × 10^7^, once)	Sodium pentobarbital and isoflurane	RESV+A549/A549, Ga	250 mg/kg, QD, 84 days	3; 5	3. *p* > 0.05; 5. *p* < 0.05
Antonio et al., 2021	Spain	BALB/c mice (Female, 10, 10/10)	20	HTT (LP07, Sc, 4 × 10^5^/200 μl, once)	Sodium pentobarbital	RESV+LP07/LP07, Ip	20 mg/kg, QD, 15 days	2; 3	2. *p* < 0.05; 3. *p* < 0.001
Savio et al. (34)	Italy	C57BL/6 J mice (Male, 4, 5/6)	NA	HTT (LLC, Sc, 1 × 10^6^/400 μL, once)	NA	RESV+LLC/LLC, Ga	125 mg/kg, QD, 21 days	1; 2; 5	1. *p* < 0.05; 2. *p* > 0.05; 5. *p* < 0.001
Liang et al. (29)	China	BALB/c mice (Male, 8–10, 5/5)	20 ± 1	HTT (A549, Sc, 5 × 10^6^/200 μl, once)	CO_2_	RESV+A549/A549, Ip	10 mg/kg, QD, 28 days	1; 2	1. *p* < 0.001; 2. *p* < 0.01

B2D, Twice every 2 days; BaP, benzo[a]pyrene; FOXC2, Forkhead Box C2; Ga, Gavage; HTT, Heterotopic tumor transplantation; Ii, Intratumor injection; Ip, Intraperitoneal; Iv, Intravenous injection; LC, Lung cancer; LLC, Lewis lung cancer; NA, Not available; NNK, 4-(N-methyl-N-nitrosamino)-1-(3-pyridyl)-1-butanone; QD, Quaque die; Q2D, Quaque secunda die; Q3D, Quaque tertia die; RESV, Resveratrol; Sc, Subcutaneous injection; SCI, Severe combined Immunodeficient; TW, Three times a week; 1, Tumor volume; 2. Tumor weight; 3. Body weight; 4. Lung metastases number; 5. Apoptotic cell proportion.

### Quality of included studies

**Figure 3:**
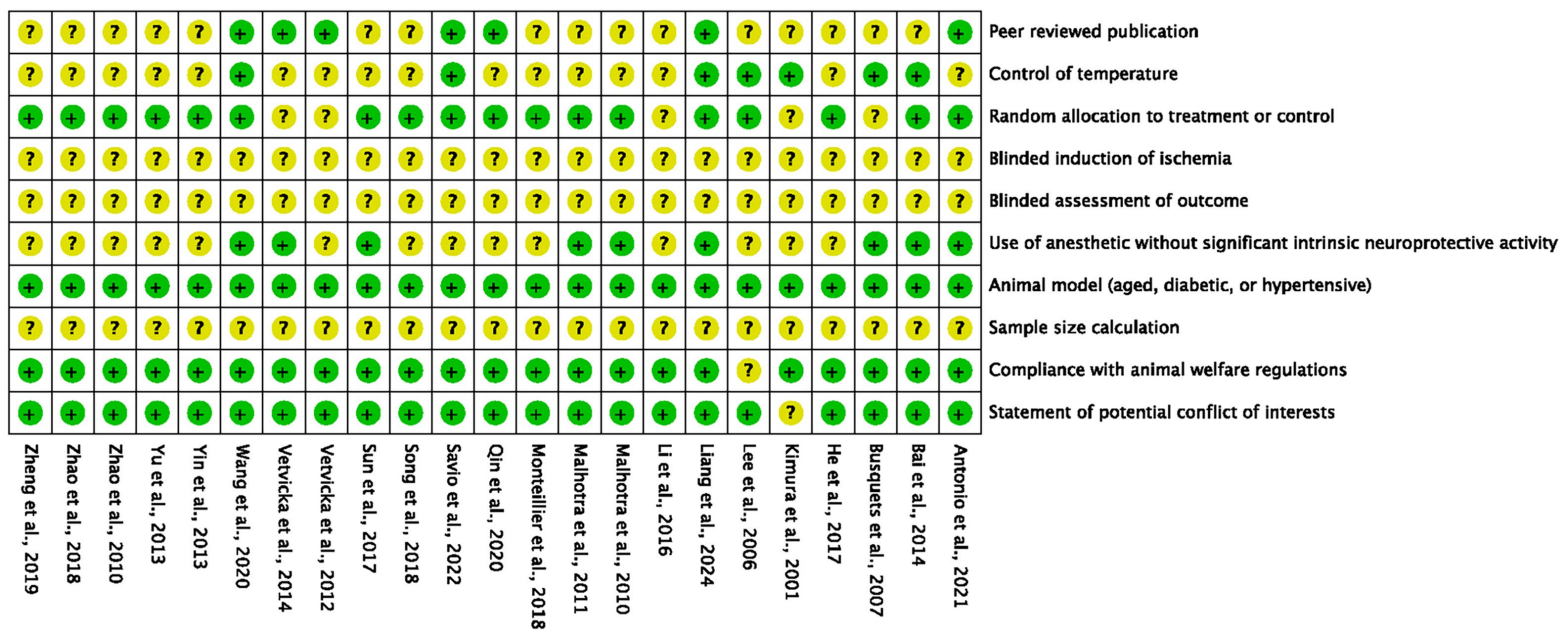
Risk of bias assessment of included studies.

According to the 10-item CAMARADES checklist, the quality scores of included studies ranged from 3 to 7, with an average score of 4.7 (Figure 3). Two studies received (29, 44) 7 points, three studies received 6 points (28, 34, 36), six studies received 5 points (17, 25, 32, 39–41), 10 studies received 4 points (23, 24, 26, 27, 31, 33, 35, 38, 42, 43), and two studies received 3 points (30, 37). All studies reported detailed information on animal models, and 22 studies reported compliance with animal welfare regulations and statements of potential conflicts of interest. However, no studies have reported the application of blindness and sample size calculation.

### Intervention effects

#### Primary outcomes

##### Tumor volume

**Figure 4:**
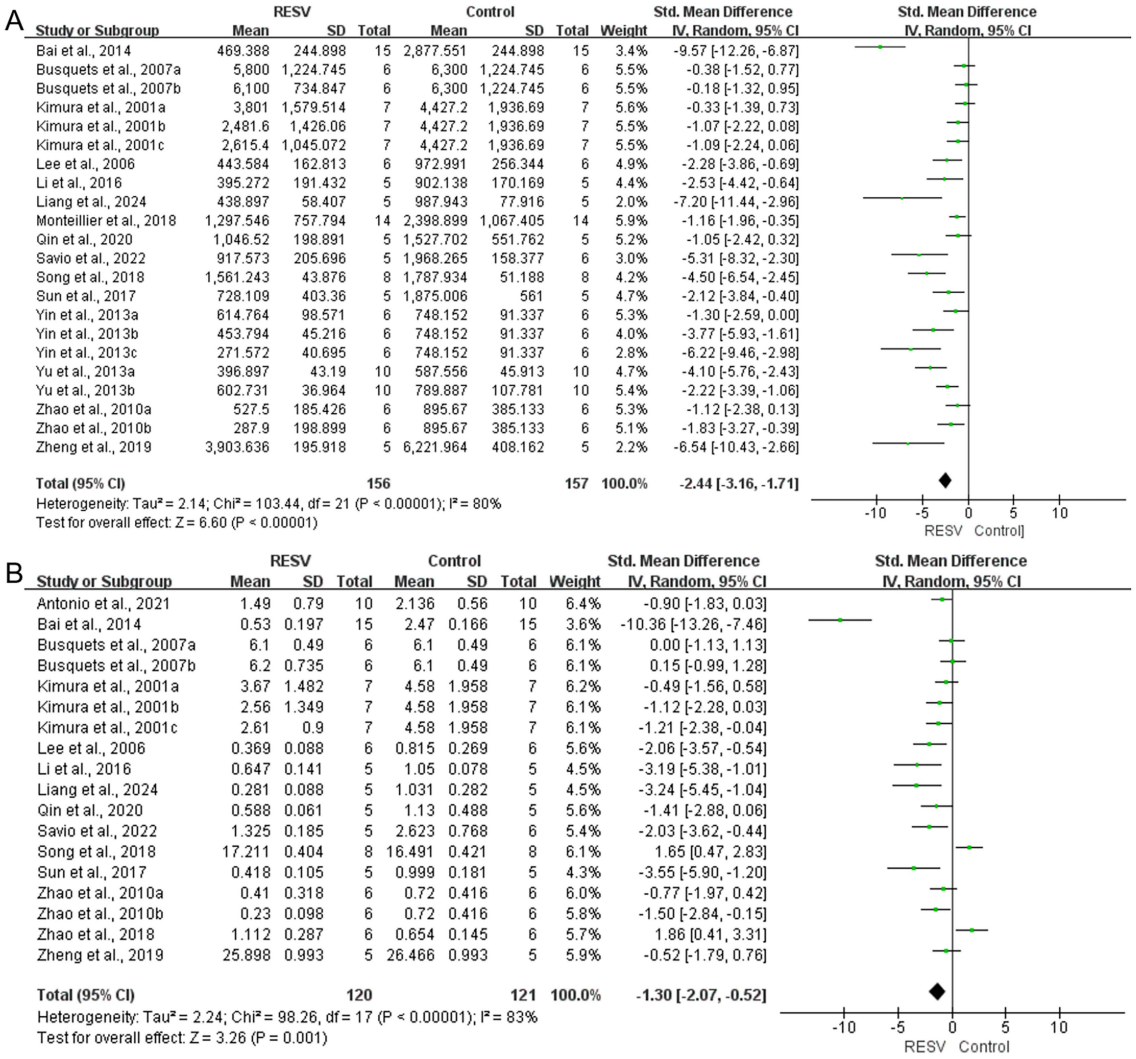
The meta-analysis result of the effect of RESV on primary outcomes of LC. **(A)** Tumor volume (mm^3^); **(B)** Tumor weight (g).

Data from 313 mice (156 in the intervention group and 157 in the control group) from 22 experiments showed that RESV significantly reduced tumor volume of LC [SMD = −2.44, 95% CI (−3.16, −1.71), *p* < 0.00001]. High heterogeneity was observed among the experiments (*I*^2^ = 80%, *p* < 0.00001; Figure 4A).

##### Tumor weight

A total of 18 experiments with 241 mice (120 in the intervention group and 121 in the control group) revealed a significant reduction of tumor weight of LC [SMD = −1.30, 95% CI (−2.07, −0.52), *p* = 0.001]. The heterogeneity analysis showed high heterogeneity among the 18 experiments (*I*^2^ = 83%, *p* < 0.00001; Figure 4B).

#### Secondary outcomes

##### Lung metastases number

**Figure 5:**
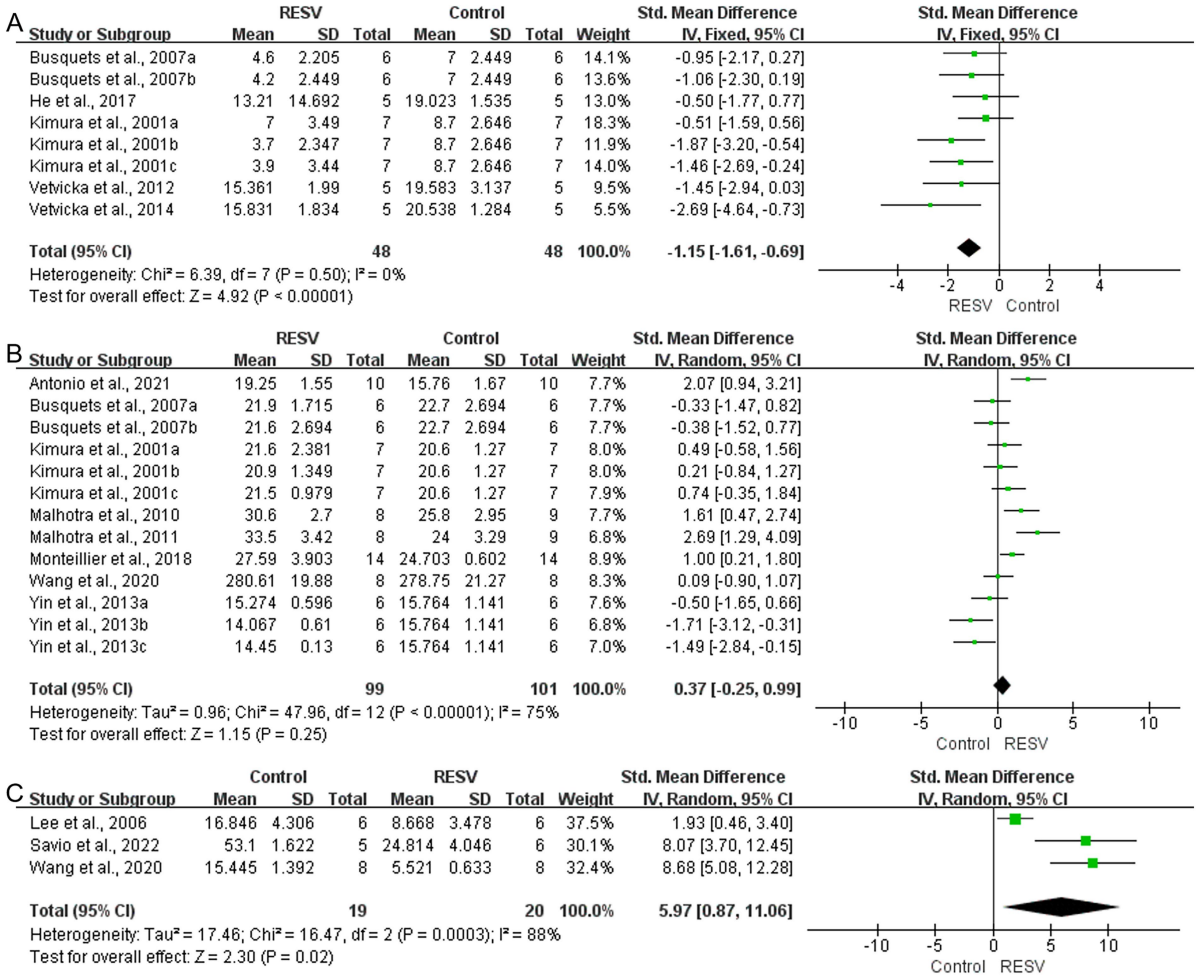
The meta-analysis result of the effect of RESV on secondary outcomes of LC. **(A)** Lung metastases number; **(B)** Body weight (g); **(C)** Apoptotic cell proportion (%).

A total of 8 experiments with 96 mice (48 in the intervention group and 48 in the control group) indicated that RESV significantly decreased the lung metastases number of LC [SMD = −1.15, 95% CI (−1.61, −0.69), *p* < 0.00001]. There was low heterogeneity among the experiments (*I*^2^ = 0%, *p* = 0.50; Figure 5A).

##### Body weight

A total of 13 experiments with 200 mice (99 in the intervention group and 101 in the control group) indicated that RESV increased the body weight of LC mice, while the difference was not statistically significant [SMD = 0.37, 95% CI (−0.25, 0.99), *p* = 0.25]. There was moderate heterogeneity among the experiments (*I*^2^ = 75%, *p* < 0.00001; Figure 5B).

##### Apoptotic cell proportion

A total of 3 experiments with 39 mice (19 in the intervention group and 20 in the control group) indicated that RESV significantly increased apoptotic cell proportion of LC cells [SMD = 5.97, 95% CI (0.87, 11.06), *p* = 0.02]. High heterogeneity was observed (*I*^2^ = 88%, *p* = 0.0003; Figure 5C).

### Exploration of heterogeneity sources

**Figure 6:**
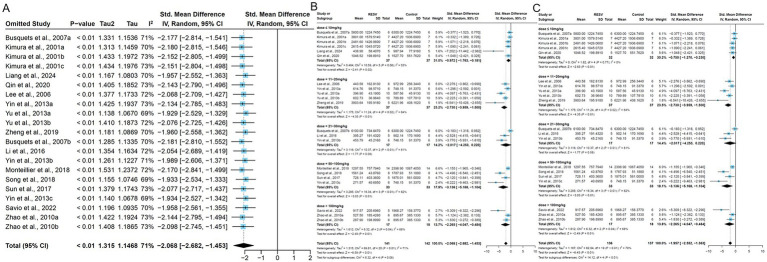
The subgroup and sensitivity analysis of RESV on tumor volume (mm^3^) of LC. **(A)** Forest plot of leave-one-out sensitivity analysis; **(B)** Forest plot of tumor volume [subgroup analysis by dose of RESV after removing Bai et al. (36)]; **(C)** Forest plot of tumor volume [subgroup analysis by dose of RESV after removing Liang et al. (29)].

Due to the observed heterogeneity across studies, we used tumor volume as the standardized measurement indicator. First, to assess the robustness of our findings, we performed a leave-one-out sensitivity analysis by sequentially excluding individual trials. The results indicated that after omitting each experiment one by one, the pooled effect estimate did not change significantly, suggesting that the observed heterogeneity could not be attributed to any specific experiment (Figure 6A). Subsequently, we performed subgroup analysis on 21 experiments [excluding Bai et al. (36), due to unreported RESV dosage] stratified by dose ranges, which reduced the overall heterogeneity from high (*I*^2^ = 80%, *p* < 0.00001) to medium (*I*^2^ = 71%, *p* < 0.01). The results showed that the heterogeneities were moderate in the ≤10 mg/kg (*I*^2^ = 53%, *p* = 0.06), 11–20 mg/kg (*I*^2^ = 64%, *p* = 0.02), and >100 mg/kg groups (*I^2^* = 71%, *p* = 0.04), and the heterogeneities were high in the 21–30 mg/kg (*I*^2^ = 81%, *p* < 0.01) and the 50–100 mg/kg groups (*I*^2^ = 82%, *p* < 0.01; Figure 6B). We observed that the heterogeneity in the ≤10 mg/kg group was mainly caused by the result of Liang et al. (29) and the heterogeneity decreased after deleting this experiment (*I*^2^ = 0%, *p* = 0.77; Figure 6C). Furthermore, we conducted meta-regression analysis to explore the potential heterogeneity source (Table 2). The results suggested that the eight factors (publication year, dose, duration, administration, modeling method, species, gender, and region) were not a source of heterogeneity (*p* > 0.05). Consequently, the observed heterogeneity across studies may be partially attributable to variations in RESV dosage.

**Table 2:** Elucidate the source of the heterogeneity using meta-regression.

Factor	*P* > |t|	95% CIs	
Publication year	1.00	−0.08	0.08
Dose	1.00	−0.22	0.22
Duration	1.00	−0.23	0.23
Administration	1.00	−0.52	0.52
Modeling method	0.39	−1.41	3.41
Species	1.00	−0.33	0.33
Sex	1.00	−0.43	0.43
Region	1.00	−0.28	0.28

### Publication bias

**Figure 7:**
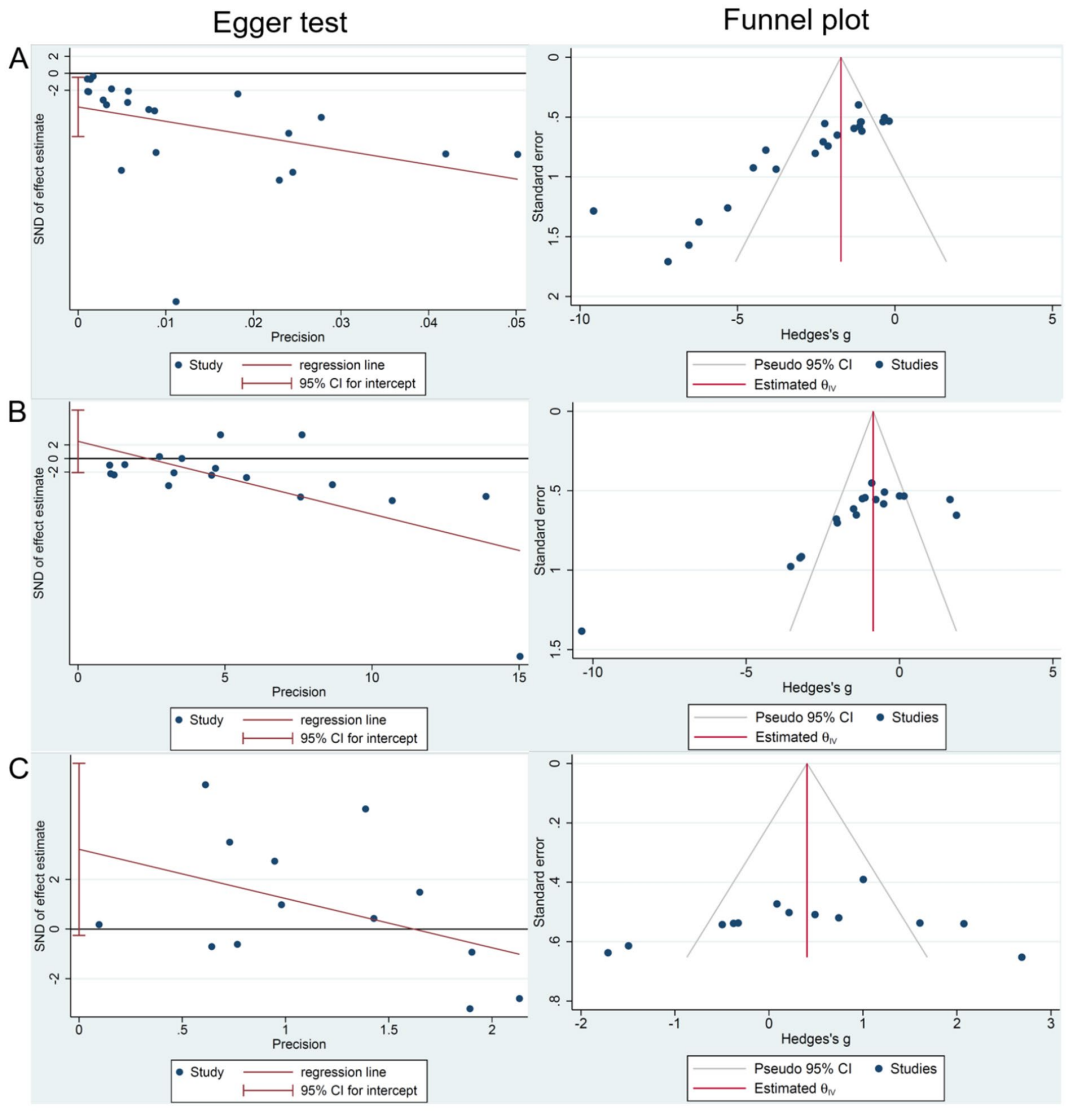
The publication bias analysis of RESV on LC. **(A)** Tumor volume (mm^3^); **(B)** Tumor weight (g); **(C)** Body weight (g).

We conducted funnel plots and Egger’s test to assess publication bias of three outcomes: (1) tumor volume; (2) tumor weight; and (3) body weight. The results showed significant publication bias in 22 studies focusing on tumor volume (*P*_egger_ = 0.028; Figure 7A), and no significant bias in 18 studies focusing on tumor weight (*P*_egger_ = 0.262; Figure 7B) and 13 studies focusing on body weight (*P*_egger_ = 0.066; Figure 7C).

### Dose–duration–effect 3D model

**Figure 8:**
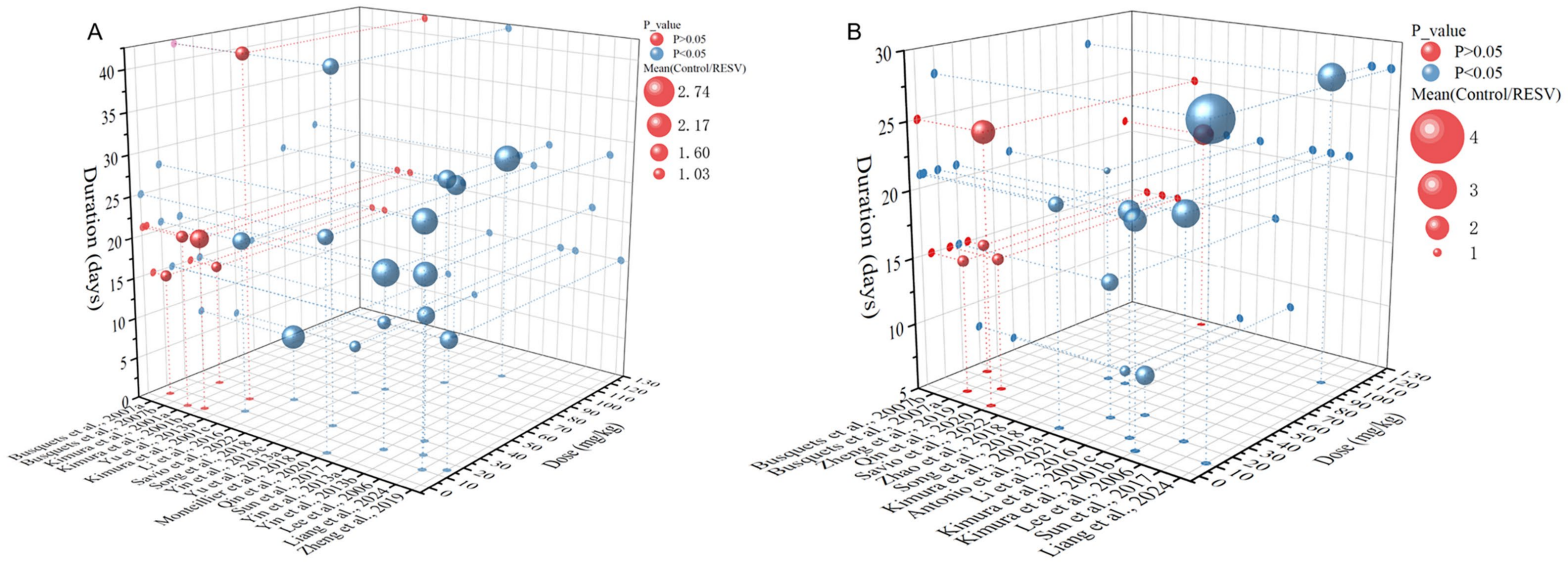
The dose–duration–effect 3D model of RESV treating LC. **(A)** Tumor volume (mm^3^); **(B)** tumor weight (g).

To identify the most effective RESV dose and duration for preclinical LC, we conducted a dose–duration–effect 3D model of primary outcomes. One study (23) with a significantly large RESV dose (1,000 and 3,000 mg/kg), which was quite different from other studies, was excluded. Additionally, one study (44) that used rats and another study (36) that reported unclear RESV dose (200 μL) were also excluded. Hence, the RESV dose used for the 3D model was from 0.23 to 125 mg/kg. The tumor volume was significantly suppressed when the dose of RESV was no less 30 mg/kg, and the treatment lasted for 25–28 days (Figure 8A). Furthermore, in studies where tumor weight was significantly reduced, the duration of RESV treatment was predominantly concentrated within 21–28 days, with doses typically ranging from 30 to 100 mg/kg (Figure 8B). Thus, the optimized dose and duration of RESV for LC were 30–100 mg/kg and 25–28 days.

### Toxicology of RESV

RESV demonstrates potential nephrotoxicity (59% probability) and cardiotoxicity (51% probability), with no other organ toxicity and no toxicity endpoint shown (Table 3). Furthermore, the predicted toxicity class was 4, and the predicted LD50 of RESV was 1,560 mg/kg, which was higher than most of the doses in available studies.

**Table 3:** Evaluation of toxicological parameters of RESV through ProTox-3.0.

Classification	Target	Prediction	Probability
Organ toxicity	Hepatotoxicity	Inactive	0.74
Organ toxicity	Neurotoxicity	Inactive	0.77
Organ toxicity	Nephrotoxicity	Active	0.59
Organ toxicity	Respiratory toxicity	Inactive	0.57
Organ toxicity	Cardiotoxicity	Active	0.51
Toxicity endpoints	Carcinogenicity	Inactive	0.71
Toxicity endpoints	Immunotoxicity	Inactive	0.86
Toxicity endpoints	Mutagenicity	Inactive	0.92
Toxicity endpoints	Cytotoxicity	Inactive	0.98
Toxicity endpoints	BBB	Inactive	0.55
Toxicity endpoints	Ecotoxicity	Inactive	0.55
Toxicity endpoints	Clinical toxicity	Inactive	0.60
Toxicity endpoints	Nutritional toxicity	Inactive	0.89

## Discussion

### Primary findings

Previous meta-analysis evaluated the effect of RESV on the incidence of LC tumors (19). However, it failed to comprehensively evaluate the efficacy and safety of RESV on LC. With the publication of a large number of preclinical studies in recent years, we conducted this updated meta-analysis to systematically evaluate the efficacy and safety of RESV for LC in multiple dimensions, including tumor volume, tumor weight, lung metastases number, body weight, and apoptotic cell proportion. Moreover, the toxicological characteristics and optimal dose and duration of RESV were explored. To be specific, 30 experiments from 23 studies were synthesized, and the quality of the included studies was moderate. RESV significantly reduced tumor volume, tumor weight, and lung metastases number, and increased the apoptotic cell proportion, while it could not improve the body weight of mice. High heterogeneities were observed in primary outcomes, and the dose of RESV may be a potential heterogeneity source. The suggested dose and duration of RESV for LC mice were 30–100 mg/kg and 25–28 days, while the LD50 of RESV was 1,560 mg/kg, indicating that RESV was safe for LC mice.

### Heterogeneity

The considerable heterogeneity observed in tumor volume may be attributable to multiple confounding variables across the included trials, including variations in experimental methodologies (modeling techniques, administration protocols, and treatment duration), pharmacological parameters (RESV dosage and formulation), and biological factors (animal species and sex), in addition to potential temporal trends reflected by publication year. *A priori*, we anticipated substantial heterogeneity given these methodological variations. Subsequent subgroup stratification by RESV dose partly reduced heterogeneity metrics, suggesting dosage variation as a partial explanatory factor. However, residual heterogeneity persisted despite comprehensive meta-regression analyses, implying the potential influence of unmeasured covariates. These may include inter-laboratory environmental differences, instrumentation variability, and technical artifacts in image-based data extraction processes.

### Potential mechanism of RESV for LC

#### Decrease the viability of the LC cells

**Figure 9:**
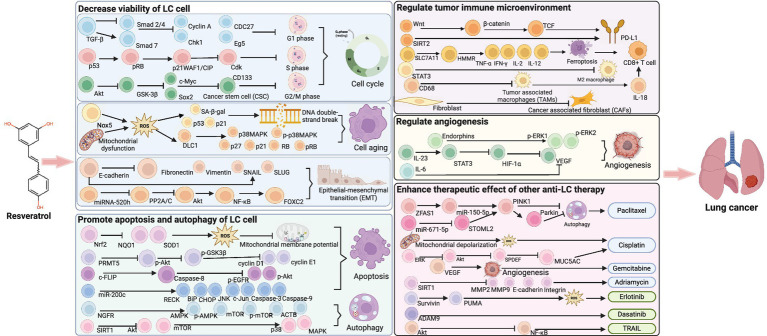
Potential mechanism of RESV for LC (created using BioRender.com).

Figure 9 shows the mechanism of RESV for LC. RESV decreased the viability of LC cells through regulating cell cycle, aging, and epithelial–mesenchymal transition (EMT). RESV maintained the A549 cell cycle in the G1 phase and altered the expression of cyclin A, Chk1, CDC27, and Eg5 (45). In another study, RESV induced a concentration-dependent stagnation of the A549 cell cycle in the S phase, which was associated with the inhibition of retinoblastoma protein phosphorylation and the upregulation of the cyclin-dependent kinase inhibitor p21WAF1/CIP (46). Furthermore, RESV induced the expression of NADPH oxidase-5 in A549 and H460 cells, which promoted reactive oxygen species (ROS) production and upregulated senescence-associated *β*-galactosidase (SA-β-gal), p53, and p21, resulting in DNA double-strand breaks of LC cells (47). In addition, RESV increased ROS in LC cells by inducing mitochondrial dysfunction, leading to the upregulation of aging-related molecules, including p38MAPK, p27, p21, and RB (48). Moreover, RESV reduced TGF-β-induced EMT by increasing E-cadherin and decreasing fibronectin, vimentin, SNAIL, and SLUG (49). Meanwhile, RESV suppressed FOXC2, a critical regulator of EMT, by influencing miRNA-520 h-mediated signaling pathways (42).

#### Promote apoptosis and autophagy of LC cell

Apoptosis and autophagy are gene-controlled programmed cell death and degradation pathways that contribute to maintaining cell homeostasis (50). RESV inhibited Nrf2, NQO1, and SOD1 in a dose-and time-dependent way to destroy the antioxidant pool and induce ROS, thereby promoting the apoptosis of LC cells (51). In addition, RESV promoted the transfer of apoptosis-inducing factor from cytoplasm to nucleus by increasing ROS and decreasing mitochondrial membrane potential, thereby promoting the apoptosis of LC cells (52). Meanwhile, RESV induced apoptosis by decreasing protein arginine methyltransferase 5 through inhibiting the Akt/GSK3β pathway (53). Furthermore, RESV activated caspase 8 and decreased c-FLIP, p-EGFR, and p-Akt in H460 cells, thereby promoting apoptosis (54). RESV increased NGFR mRNA expression and prolonged NGFR mRNA and protein survive, then activated AMPK-mTOR pathway to promote autophagy and apoptosis of A549 cells (55). Additionally, RESV inhibited the Akt/mTOR pathway and activated the p38-MAPK pathway by upregulating SIRT1, thereby inducing apoptosis and autophagy of LC cells (56).

#### Regulate tumor immune microenvironment

The heterogeneity of the immune microenvironment is related to the progression and treatment responsiveness of cancer. RESV significantly increased the expression of programmed cell death ligand 1 in NCI-H358 cells, which plays a vital role in suppressing T-cell-mediated immune responses (57). Triacetylresveratrol, a RESV analog, is a potent SIRT2 agonist and is associated with the infiltration of multiple immune cells in LC, including CD8 + T cells, CD4 + T cells, CD4 + memory resting, regulatory T cells, and natural killer cells (58). Furthermore, RESV enhanced the cytotoxic of CD8 + T cells, the most important cytotoxic immune cells, by modulating the SLC7A11-HMMR interaction and activating ferroptosis (59). Moreover, RESV downregulated STAT3 *in vitro*, thereby inhibiting M2-like polarization of tumor-associated macrophages (TAMs) and suppressing the proliferation of LC cells (17). In another study, TAM (CD68+) infiltration in LC tumors, including tumor-promoting M2 macrophages (CD163+) and lymphocytes (CD3+), was significantly reduced after RESV treatment (34). In addition, RESV promoted the secretion of IL-18 by regulating TAMs in LC, and IL-18 was a key cytokine that promoted the activation of CD8 + T cells (60). RESV prevented the transformation of normal fibroblasts into cancer-associated fibroblasts (CAFs) through regulating autophagy, thus disrupting the promotion of LC by CAFs (34).

#### Regulate angiogenesis

Angiogenesis is a crucial mechanism by which tumor cells acquire nutrients and metastasize. However, angiogenesis has been proven to be beneficial for enhancing the efficacy of anti-LC treatment. For instance, RESV reduced endorphins and increased phosphorylated ERK 1/2, thereby promoting microvessel growth and tumor blood perfusion. Under such conditions, the cytotoxic effect of gemcitabine on LC cells was significantly enhanced (39). In H460 cells, RESV downregulates VEGF expression, which is an important regulator of microvascular production (54). In another study, RESV regulated angiogenesis by inhibiting the STAT3/HIF-1α/VEGF pathway (44). In addition, RESV significantly inhibited the secretion of cytokines IL-6 and VEGF in co-cultured A549 cells and mesenchymal stem cells (61). *In vivo* studies showed that RESV reduced the expression of the angiogenic marker CD31 in tumor tissues (34). In small-cell LC, IL-23-induced inflammatory microenvironment activated the STAT3/VEGF pathway, whereas RESV inhibited this activation (62).

#### Enhance the therapeutic effect of other anti-LC therapies

A large number of studies showed that RESV was a promising anti-LC adjuvant. RESV inhibited autophagy of A549 cells by regulating ZFAS1/miR-150-5p/PINK1 pathway, thereby enhancing the sensitivity of LC to paclitaxel (63). Meanwhile, RESV enhanced the susceptibility of A549 cells to paclitaxel through miR-671-5p-mediated inhibition of STOML2 (64). RESV enhanced the effect of cisplatin on mitochondrial apoptosis of small-cell LC by promoting mitochondrial depolarization and ROS production (65). RESV regulated the SPDEF-MUC5AC axis by inhibiting ERK and Akt signaling, thereby increasing the sensitivity of LC to cisplatin (10). Moreover, RESV enhanced the inhibitory effect of gemcitabine on tumor growth by promoting tumor microvascular growth (39). Meanwhile, RESV decreased SIRT1, MMP2, MMP9, E-cadherin, and integrin in A549 cells to enhance the cytotoxic of adriamycin (66). RESV induced ROS production by downregulating survivin and upregulating PUMA, thereby promoting erlotinib-mediated LC cell apoptosis (67). RESV promoted ADAM9 degradation in LC cells through the ubiquitin–proteasome pathway, thereby enhancing the therapeutic effect of dasatinib on LC cells (68). In A549 and HCC-15 cells, RESV reduced LC resistance to tumor necrosis factor-related apoptosis-inducing ligand by inhibiting the Akt/NF-κB pathway (69). Finally, RESV increased the sensitivity of LC to radiotherapy by upregulating ROS and SA-*β*-gal in A549 and H460 cells and promoting DNA double-strand breaks (70).

#### Limitations

Several noteworthy limitations should be acknowledged in this study. First, the generalizability of our findings is constrained by substantial heterogeneity and moderate-to-low methodological quality across included studies. While subgroup analyses and meta-regression identified RESV dosage as a potential source of heterogeneity, residual variability may be explained by additional confounding factors such as experimental conditions and measurement instrumentation. Second, the toxicity predictions generated by ProTox-3.0 require empirical validation through experimental approaches, such as histopathological examination and serum biomarker analysis in preclinical models. Third, despite demonstrating promising clinical potential, the therapeutic application of RESV remains limited by its unfavorable pharmacokinetic properties, notably poor oral bioavailability and rapid systemic metabolism, which pose significant challenges for clinical translation.

#### Mechanistic investigation and clinical translation challenges of RESV in LC treatment

The mechanistic underpinnings of RESV in LC treatment necessitate further preclinical investigation. Notably, the therapeutic efficacy of RESV against LC is modulated by multiple variables, including dosage, duration, and administration route. Herein, we systematically evaluated the interrelationships among quantifiable parameters (dose, duration, and therapeutic effect) to establish the potentially optimal dosage and duration of RESV in LC-bearing mouse models. The administration route was excluded due to its inherent non-quantifiable nature and inability to account for heterogeneity sources. However, subsequent researches are encouraged to comprehensively apply our findings to explore more appropriate administration routes, which would significantly contribute to systematically optimizing preclinical protocols for RESV-based LC therapy. More importantly, there are other crucial issues that must be addressed prior to clinical translation. RESV exhibits rapid metabolic clearance in humans, undergoing extensive first-pass metabolism in the liver and intestine, where it is converted to inactive sulfate conjugates, resulting in notably low systemic bioavailability (16). To optimize RESV’s anti-LC efficacy in human applications, key pharmacological parameters, including formulation, dosage regimen, and administration route, require systematic optimization. While our study identified 30–100 mg/kg as the optimal dose range in animal models, subsequent studies must establish the human equivalent dose through appropriate scaling methods. Furthermore, since mouse models are unable to fully replicate the human tumor microenvironment or pharmacokinetics, it must be recognized that the optimal dose and duration proposed in this study cannot simply be applied to human research through equivalent dose conversion. Previous clinical investigations have demonstrated that micronized formulations can enhance RESV bioavailability by approximately threefold compared to conventional preparations, achieved through increased surface area and improved suspension properties (71). Current preclinical research has focused extensively on RESV nanoparticle development for LC therapy. These engineered formulations improve bioavailability through multiple mechanisms, such as enhancing aqueous solubility, improving chemical stability, controlling release kinetics, and targeted delivery (16). Although still in preclinical development, RESV nanoparticles demonstrate promising translational potential. While clinical evaluation of RESV for LC treatment represents a crucial next step, rigorous resolution of these pharmacological challenges is imperative to ensure patient safety and therapeutic efficacy. The transition to human trials must be predicated on comprehensive preclinical data addressing these fundamental issues.

## Conclusion

As a safe food-derived compound, RESV can effectively inhibit LC tumor growth *in vivo*, especially to control tumor volume, tumor weight, lung metastasis number, and tumor cell apoptosis rate. Its action mechanism is complex and may be related to regulating cell cycle, cell senescence, EMT, apoptosis, autophagy, angiogenesis, and immune microenvironment. However, the clinical research on RESV treatment of LC is limited, which is the key research field to promote the clinical application of RESV.

## Data Availability

The original contributions presented in the study are included in the article/Supplementary material, further inquiries can be directed to the corresponding authors.

## Footnotes

^1^ https://automeris.io/login/

^2^ https://comptox.charite.de/protox3/

## Glossary

B2D: twice every 2 days
BaP: benzo[a]pyrene
BBB: blood–brain barrier
CAFs: cancer-associated fibroblasts
CI: confidence interval
EMT: epithelial–mesenchymal transition
FOXC2: forkhead box c2
Ga: gavage
HTT: heterotopic tumor transplantation
Ii: intratumor injection
Ip: intraperitoneal
Iv: intravenous injection
LC: lung cancer
LD50: median lethal dose
LLC: lewis lung cancer
NA: not available
NNK: 4-(N-methyl-N-nitrosamino)-1-(3-pyridyl)-1-butanone
Q2D: quaque secunda die
Q3D: quaque tertia die
QD: quaque die
RESV: resveratrol
ROS: reactive oxygen species
SA-*β*-gal: senescence-associated β-galactosidase
Sc: subcutaneous injection
SCI: severe combined Immunodeficient
SMD: standardized mean difference
TAMs: tumor-associated macrophages
TW: three times a week
WMD: weighted mean difference
