# Supplementary material for: Therapeutic effects and safety of resveratrol for lung cancer: an updated preclinical systematic review and meta-analysis
Source: Front Nutr. 2025 Aug 29;12:1644538. doi: 10.3389/fnut.2025.1644538 (PMC12425900; doi:10.3389/fnut.2025.1644538)
Supplement: Supplementary file 1 [file Table_1.DOCX]

**Search strategy**

**Datasets: PubMed, Embase, Web of Science, Cochrane Library**

**Search date: 2024-07-14**

**PubMed (364 articles):**

((Lung cancer) OR (Carcinoma, Non-Small-Cell Lung) OR (Carcinoma, Non Small Cell Lung) OR (Carcinomas, Non-Small-Cell Lung) OR (Lung Carcinoma, Non-Small-Cell) OR (Lung Carcinomas, Non-Small-Cell) OR (Non-Small-Cell Lung Carcinomas) OR (Carcinoma, Non-Small Cell Lung) OR (Non-Small Cell Lung Cancer) OR (Non-Small-Cell Lung Carcinoma) OR (Non Small Cell Lung Carcinoma) OR (Nonsmall Cell Lung Cancer) OR (Non-Small Cell Lung Carcinoma) OR (Adenocarcinoma of Lung) OR (Lung Adenocarcinomas) OR (Lung Adenocarcinoma) OR (Adenocarcinoma, Lung) OR (Adenocarcinomas, Lung) OR (Squamous cell carcinoma of lung) OR (lung squamous cell carcinoma)) AND ((Resveratrol) OR (3,4',5-Stilbenetriol) OR (3,5,4'-Trihydroxystilbene) OR (3,4',5-Trihydroxystilbene) OR (trans-Resveratrol) OR (trans Resveratrol) OR (Resveratrol-3-sulfate) OR (Resveratrol 3 sulfate) OR (SRT 501) OR (SRT-501) OR (SRT501) OR (cis-Resveratrol) OR (cis Resveratrol) OR (Resveratrol, (Z)-) OR (trans-Resveratrol-3-O-sulfate) OR (trans Resveratrol 3 O sulfate))

**Embase (1,366 articles):**

((Lung cancer) OR (Carcinoma, Non-Small-Cell Lung) OR (Carcinoma, Non Small Cell Lung) OR (Carcinomas, Non-Small-Cell Lung) OR (Lung Carcinoma, Non-Small-Cell) OR (Lung Carcinomas, Non-Small-Cell) OR (Non-Small-Cell Lung Carcinomas) OR (Carcinoma, Non-Small Cell Lung) OR (Non-Small Cell Lung Cancer) OR (Non-Small-Cell Lung Carcinoma) OR (Non Small Cell Lung Carcinoma) OR (Nonsmall Cell Lung Cancer) OR (Non-Small Cell Lung Carcinoma) OR (Adenocarcinoma of Lung) OR (Lung Adenocarcinomas) OR (Lung Adenocarcinoma) OR (Adenocarcinoma, Lung) OR (Adenocarcinomas, Lung) OR (Squamous cell carcinoma of lung) OR (lung squamous cell carcinoma)) AND ((Resveratrol) OR (trans Resveratrol) OR (Resveratrol 3 sulfate) OR (SRT 501) OR (SRT501) OR (cis Resveratrol) OR (trans Resveratrol 3 O sulfate))

**Web of Science (529 articles):**

#1: (((TS=(Lung cancer)) OR TS=(Non-Small Cell Lung Cancer)) OR TS=(Lung Adenocarcinoma)) OR TS=(Lung squamous cell carcinoma)

#2: (((((TS=(Resveratrol)) OR TS=(trans Resveratrol)) OR TS=(Resveratrol 3 sulfate)) OR TS=(SRT 501)) OR TS=(cis Resveratrol)) OR TS=(trans Resveratrol 3 O sulfate)

#3: #1 AND #2

**Cochrane Library (2 articles):**

#1: (lung cancer):ti,ab,kw OR (Non-Small Cell Lung Cancer):ti,ab,kw OR (Lung Adenocarcinoma):ti,ab,kw OR (Lung squamous cell carcinoma):ti,ab,kw

#2: (Resveratrol):ti,ab,kw OR (trans Resveratrol):ti,ab,kw OR (Resveratrol 3 sulfate):ti,ab,kw OR (SRT 501):ti,ab,kw OR (cis Resveratrol):ti,ab,kw

#3: #1 AND #2
